# Supplementary material for: Gain-switched semiconductor laser driven soliton microcombs
Source: Nat Commun. 2021 Mar 3;12:1425. doi: 10.1038/s41467-021-21569-7 (PMC7930029; doi:10.1038/s41467-021-21569-7)
Supplement: Supplementary file 1 — Supplementary Information [file 41467_2021_21569_MOESM1_ESM.pdf]

Supplementary Information for:
Gain-switched semiconductor laser driven soliton microcombs

Weng and Kaszubowska-Anandarajah et al.

SUPPLEMENTARY NOTE 1: GAIN-SWITCHING LASERS AND LASER BIAS CURRENT

Supplementary Figure 1 shows the three semiconductor lasers used in this study. The DFB laser and the FP laser, when gain-switched, can be operated in either the single-mode lasing mode or the dual-mode lasing mode with proper tuning of the master laser wavelength. The DM laser, on the other hand, can only work in single-mode lasing mode due to the strong suppression of the side modes.

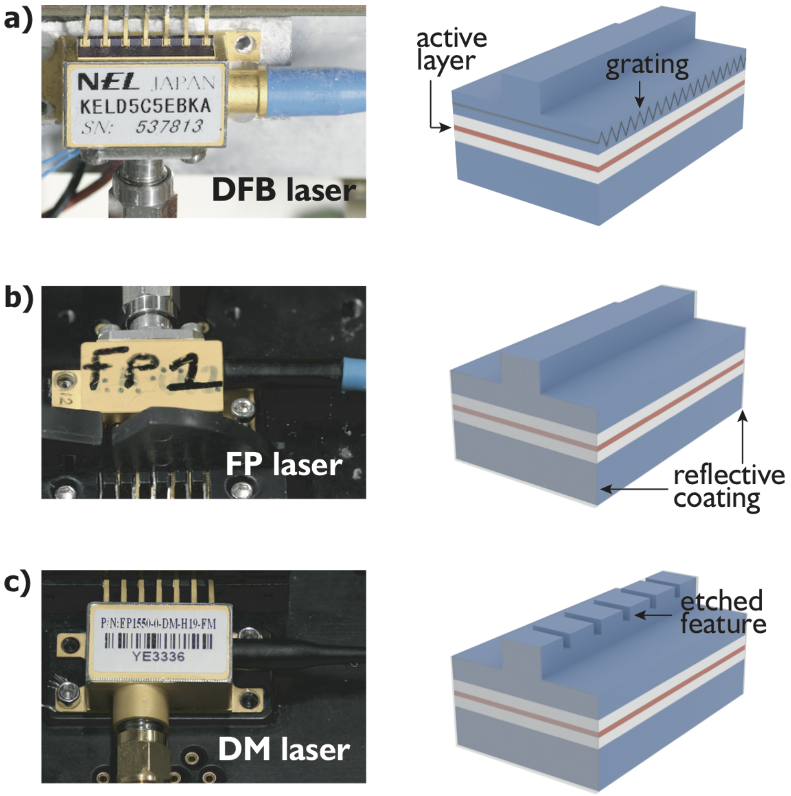

Supplementary Figure 1. **Three semiconductor lasers used in this study.** The photographs (left-hand side panels) and the laser structure illustrations (right-hand side panels) of (a) the DFB laser, (b) the FP laser, and (c) the DM laser.

In Supplementary Figure 2 we show the measured DM laser bias current at the output of the bias tee at three different microwave power levels, respectively. We keep the dc current to be constant at 50 mA, and vary the ac power from 20 to 24 dBm. The laser impedance is 50 ohms. We note that a large portion of the microwave power is dissipated by a resistor in the laser package for impedance matching. In the future with optimised rf circuits design the consumed microwave power may be significantly lowered.

SUPPLEMENTARY NOTE 2: COMPARISON BETWEEN MODELS BASED ON SINGLE-MODE AND DUAL-MODE LASER RATE EQUATIONS

To numerically investigate the dynamics of single-mode-lasing gain-switched lasers (e. g., when the GSL is central-injection-locked, or when the single-mode DM laser is used), conventional single-mode rate equations are adequate to describe the coupling between the lasing field and the carrier density. The equations can be written as:

$$\frac{dN}{dt} = \frac{I_{\mathrm{bias}} + I_{\mathrm{gs}} \sin(2\pi f_{\mathrm{gs}} t)}{eV} - (\gamma_1 N + \gamma_2 N^2 + \gamma_3 N^3) - aV(N - N_0)|A_{\mathrm{GSL}}|^2 + F_{\mathrm{N}} \tag{1}$$

$$\frac{dA_{\mathrm{GSL}}}{dt} = \frac{1}{2}(1 - i\alpha_{\mathrm{H}}) \left(aV(N - N_0) - \frac{1}{\tau_{\mathrm{p}}} \right) A_{\mathrm{GSL}} + \sqrt{\kappa_{\mathrm{c}}} s_{\mathrm{inj}} + F_{\mathrm{A}} \tag{2}$$

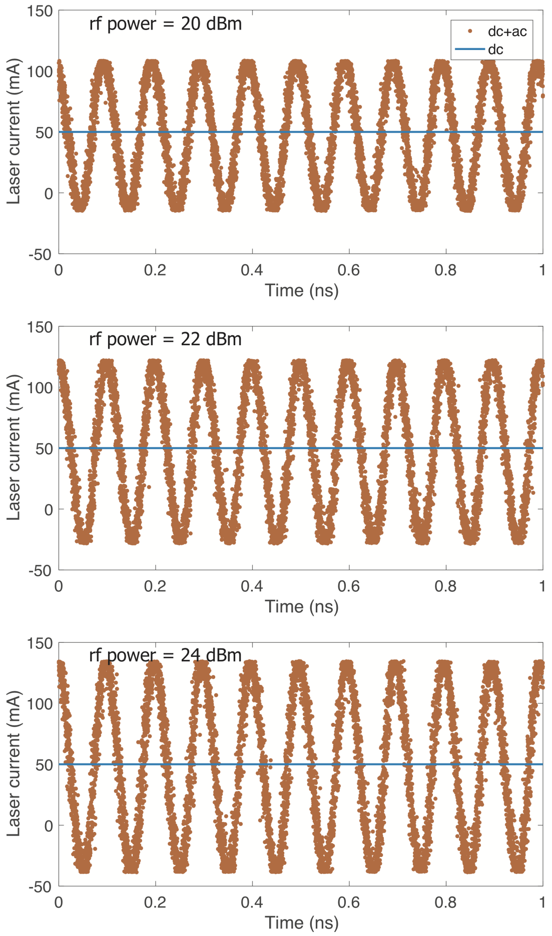

Supplementary Figure 2. **Measured current output from the bias tee in the DM laser.** The applied rf power levels are 20 dBm (top), 22 dBm (middle), and 24 dBm (bottom), respectively. The impedance of the laser is 50 ohms.

We compare the results derived from the single-mode rate equations and those derived with the dual-mode rate equations in the main text. All the parameter values used in the simulation based on the single-mode rate equations are the same as those for the model based on the dual-mode rate equations. Supplementary Figure 3 shows the simulated lasing spectra: (a, b) CW lasing without master laser injection; (c, d) GSL without injection; and (e, f) central-injection-locked GSL. The left-hand panels are based on single-mode laser rate equations, while the right-hand panels are obtained with dual-mode rate equations. These comparisons show that the single-mode model and the dual-mode model yield nearly identical results. Despite that the dual-mode model has two modes, only the central mode at 1549 nm with a longer photon lifetime (i. e., less cavity loss) outputs power effectively. Such a result is a typical winner-takes-all case. When two modes compete with each other for lasing resources (i. e., carriers), unless external injection is strongly partial to the disadvantaged mode, the one with a higher gain or a lower loss would drain all the carriers. It is also clear that with the master injection the noise floors in the GSL comb spectra are substantially suppressed, showing that the injection locking improves the pulse-to-pulse coherence and narrows the comb teeth linewidths.

SUPPLEMENTARY NOTE 3: SIMULATION OF MULTIMODE LASING

In this section we provide more details on the simulation of multimode GSL with offset-injection. Dual-mode rate equations elaborated in the main text are used to obtain the results shown here. The parameter values used here are the same as those used in the main text for multimode lasing, except that the spontaneous emission effect is excluded for clarity. The side mode frequency and the master laser frequency are lower than that of the central mode by 250 GHz and 160 GHz respectively. Supplementary Figure 4 (a) presents the GSL intensity and the applied current

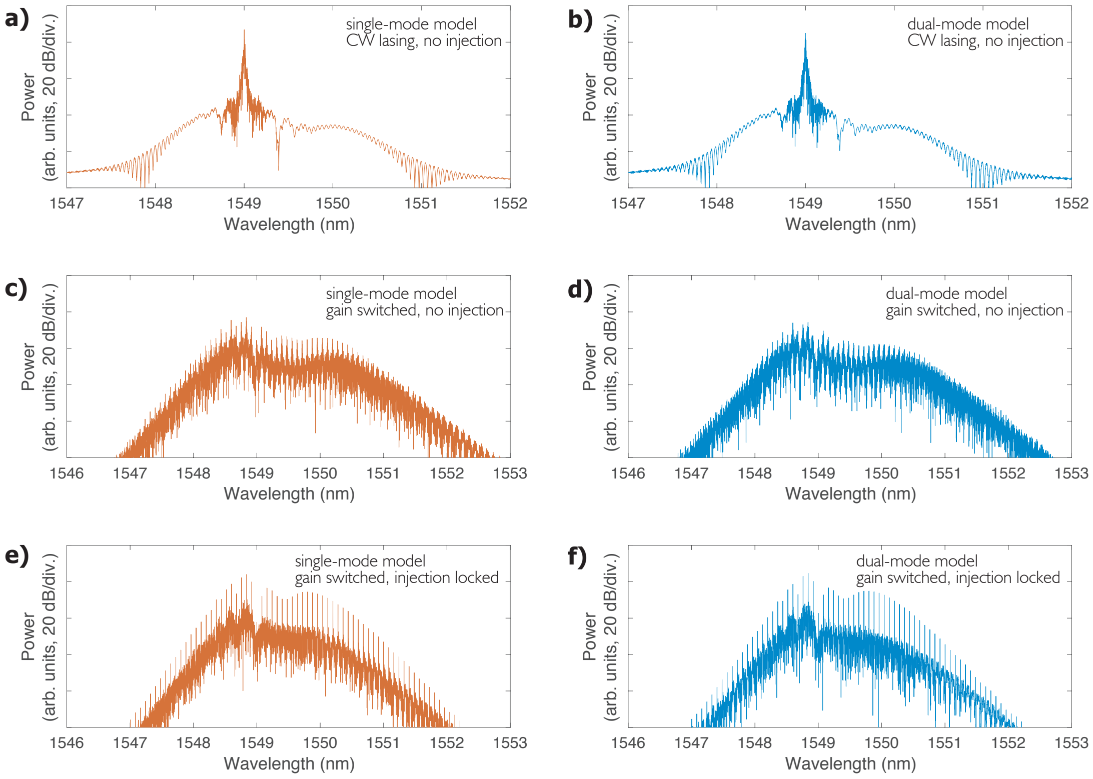

Supplementary Figure 3. **Simulated laser spectra based on laser rate equations.** (a, b) CW lasing spectra when there is no master laser injection. (c, d) GSL spectra without the injection of the master laser. (e, f) GSL spectra when the master laser is injected in the centre of the mode at 1549 nm. Left-hand panels: results obtained with the single-mode model. Right-hand panels: results numerically computed with the dual-mode model.

($I_{\text{bias}}+I_{\text{gs}}$) during 4 gain switching periods. Supplementary Figure 4 (b) shows the field amplitudes of $A_{\text{GSL}} = A_1+A_2$, A_1 (the central mode), and A_2 (the side mode) respectively. One can see that the interference between A_1 and A_2 significantly facilitates the multi-peak intensity profile of the GSL pulses. Supplementary Figure 4 (c) plots the comb spectra of the central mode and the side mode respectively, which not only shows that the dual-mode lasing broadens the GSL comb bandwidth, but also provides a straightforward way to understand the formation of the multi-peak pulses. The central mode and the side mode output fields that overlap with each other spatiotemporally. The carrier frequencies of the two fields are close to, but slightly less than the frequency difference between the two modes due to the master laser injection. As a result, the temporal gaps between adjacent peaks, which are corresponding to the carrier frequency difference, are always wider than the time scale that corresponds to the mode frequency difference. Take the offset-injection-locked DFB GSL experimental results for instance, the inter-peak separation of 4.7 ps is slightly longer than the 4-ps characteristic time as per the mode frequency difference of 250 GHz, showing excellent agreement with our analysis.

SUPPLEMENTARY NOTE 4: MICROCOMB GENERATION WITH REVERSED DETUNING SWEEPING

By adjusting the polarisation state of the master laser and the bias current, we can fine-adjust the GSL pulses to demonstrate microcomb generation and comb state switching in both pump laser frequency-up and -down sweeps. Supplementary Figure 5 (a) shows the generated microcomb power during the bidirectional sweeping with an average pump power of 15 mW. While the conventional frequency-down swept trace shows that the dual-soliton step and single-soliton step appearing subsequently after the MI stage, the frequency-up swept trace also exhibits these distinct microcomb states in a reversed order. Recently, such soliton generation with bidirectional sweeping has been demonstrated with chirped CW laser [1], showing that solitons can be formed from the condensation of an extended pattern with suitable detuning and power. Here, owing to the low pump power and consequently the reduced thermal

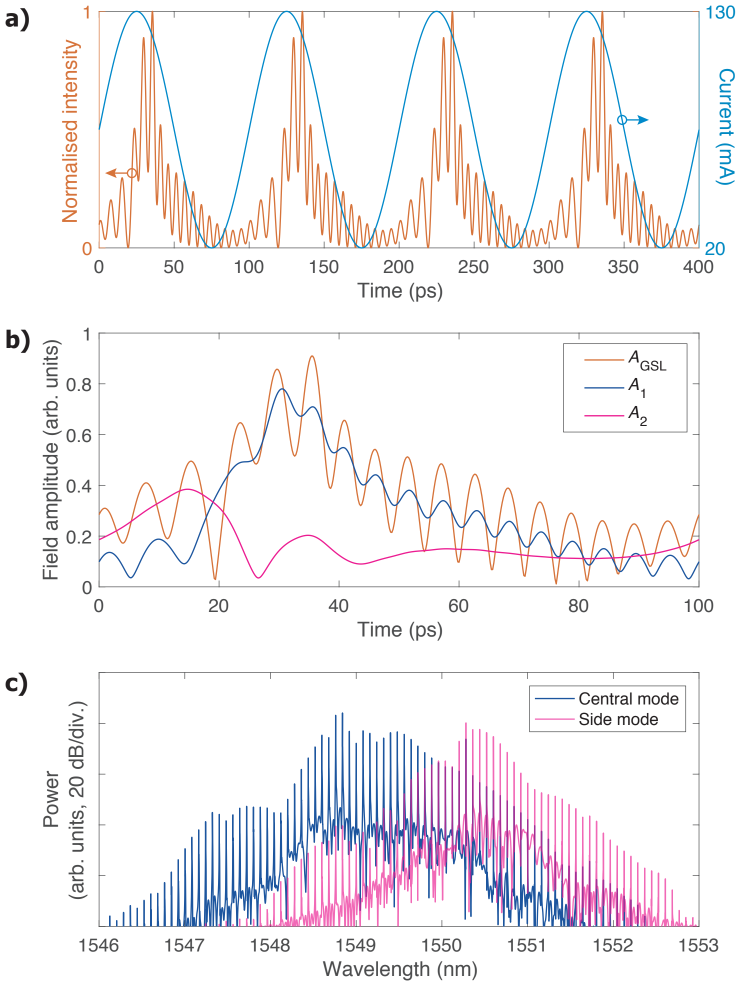

Supplementary Figure 4. **Simulation of offset-injection-locked GSL based on dual-mode rate equations.** (a) The GSL intensity and the applied current during four gain switching periods. (b) The amplitudes of the GSL field, the central mode field and the side mode field, respectively. (c) The GSL spectra of the central mode field and the side mode field, respectively.

nonlinearity, we can switch the system into any of the microcomb states in Supplementary Figure 5 (b) by manually tuning the frequency of the master laser, which promotes the convenient control and the flexible switching of microcombs. We note that, a 4.6-ps intersoliton separation in the dual-soliton state can be inferred from the comb spectrum. This separation is close to the 4.7-ps inter-peak separation measured with autocorrelation, showing that the two solitons are supported by two neighbouring peaks with local phase maxima in the GSL pulses. To corroborate the experiments, we also simulate the MI comb state, whose spectrum and temporal profile are presented in Supplementary Figure 5 (c) and (d), respectively. Our simulation shows that this MI state is coherent (i. e., chaos free).

The LLE model, with a detuning that is swept towards a higher frequency, is used to simulate the reverse sweeping in microcomb generation. To qualitatively reproduce the experimental observation in which a dual-soliton state appears after the single-soliton state, we modify the input GSL pulse profile in such a way that only two peaks are kept and their relative intensities are slightly adjusted. We note that due to the complex nature of the laser rate equations and the many nonlinear effects that are ignored in the dual-mode model, to reproduce a highly similar profile with the numerical simulation based on rate equations is not trivial. While experimentally the GSL pulse profile can be adjusted by empirical means, such as adjusting the injection polarisation and power, the temperature, the bias current as well as the gain-switching current level, the main purpose of the simulation is to show that solitons can be initiated with pulses whose carrier frequency is scanned upwards. Supplementary Figure 5 (e) shows the evolution of the intracavity power, including the pump profile on the righthand side. Despite the discrepancy in the soliton step length, the simulation demonstrates the sequential appearance of single-soliton state, dual-soliton state, and then the MI state.

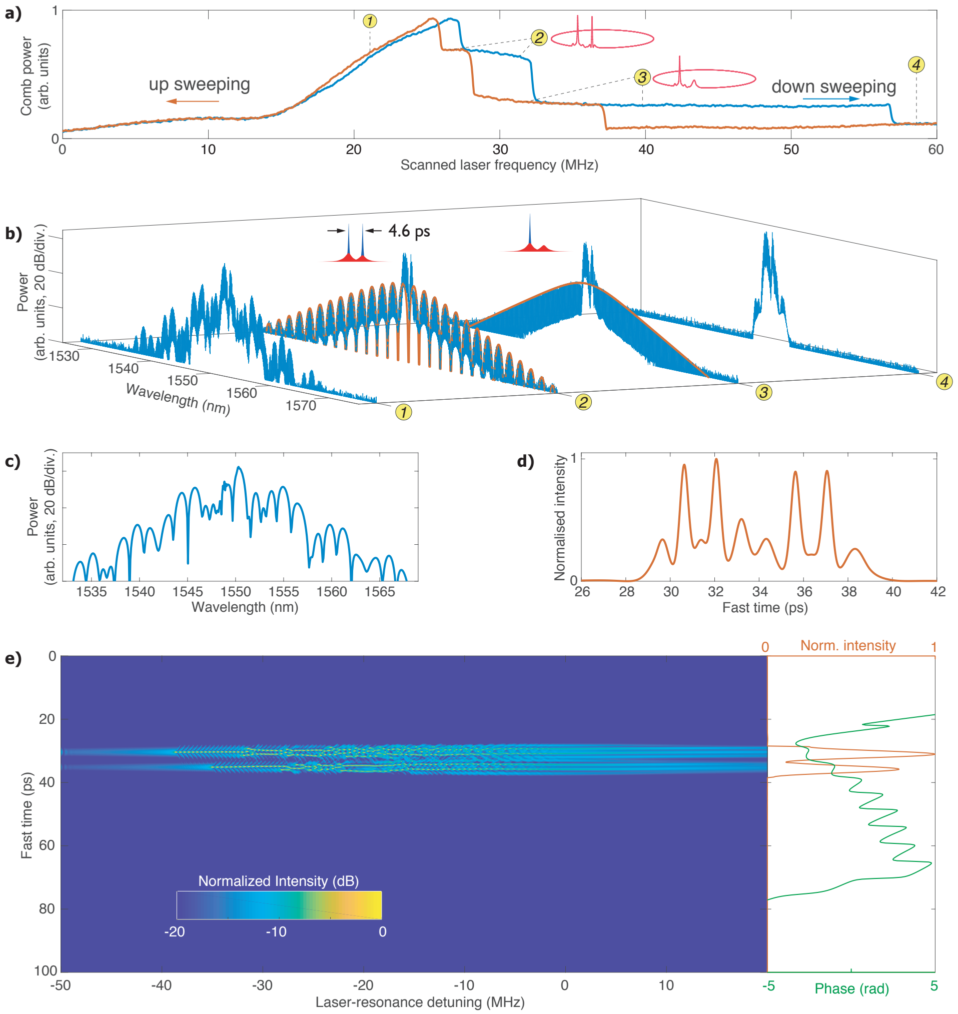

Supplementary Figure 5. **Microcomb generation with gain-switched laser reverse sweeping.** (a) Comb power spectra with the DFB GSL sweeping over resonances towards higher (red trace) and lower (blue trace) frequencies, respectively. (b) The four representative comb spectra corresponding to the four labeled positions in (a). The dual-soliton state shows a intersoliton separation of 4.6 ps. (c) Simulated microcomb spectrum that corresponds to the MI state. (d) The temporal profile of the intracavity intensity of the simulated MI state. (e) Microcomb generation simulation using frequency-up-swept pulses. On the righthand side are the intensity and phase profiles of the input pumping source. The average pumping power is 5 mW.

SUPPLEMENTARY REFERENCES

[1] Cole, D. C. *et al.* Kerr-microresonator solitons from a chirped background. *Optica* **5**, 1304–1310 (2018). URL https://www.osapublishing.org/optica/abstract.cfm?uri=optica-5-10-1304.
